# Coming Out to Parents in Lesbian and Bisexual Women: The Role of Internalized Sexual Stigma and Positive LB Identity

**DOI:** 10.3389/fpsyg.2020.609885

**Published:** 2020-12-08

**Authors:** Roberto Baiocco, Jessica Pistella, Mara Morelli

**Affiliations:** ^1^Department of Developmental and Social Psychology, Sapienza University of Rome, Rome, Italy; ^2^Department of Dynamic and Clinical Psychology, Faculty of Medicine and Psychology, Sapienza University of Rome, Rome, Italy

**Keywords:** coming out, parents, sexual orientation, bisexuality, women, internalized sexual stigma, positive identity

## Abstract

The experience of “coming out” (CO) to parents is often a crucial event in the lives of lesbian and bisexual (LB) women, associated with lower internalized sexual stigma (ISS) and higher positive LB identity. Few studies have compared the experiences of LB women in the CO process. Rather, most prior research has either: (1) not addressed bisexuality or eliminated bisexual individuals from the analysis; (2) combined bisexual women and bisexual men in the same sexual orientation group; or (3) examined bisexual participants alongside lesbian women and gay men, using a single monolithic measure. Thus, the present research aimed at investigating the role of ISS and positive LB identity in inhibiting or encouraging CO to parents in a sample of 241 lesbian women (*M*_age_ = 27.61, *SD* = 7.19) and 186 bisexual women (*M*_age_ = 25.23, *SD* = 5.81), aged 18–40 years. Most participants reported that they had already revealed their sexual orientation to their mother (69%) and their father (52%). More lesbian women had CO to both their mother and their father than had bisexual women. These lesbian women reported lower levels of ISS and higher levels of LB positive identity relative to bisexual women. On average, CO to mothers occurred at age 20 (*SD* = 5.54), while CO to fathers occurred at age 22 (*SD* = 5.63). LB women did not differ in the average age of CO to mothers or fathers, or in parental reactions to CO. Finally, ISS was found to affect the process of CO to both parents via positive identity (bootstrapping estimate = −0.26, SE = 0.08, 95% CI = −0.43, 0.11), whereas sexual orientation was not found to moderate the path from ISS to CO to both parents. The present study contributes to our understanding of the differences between LB women when developing their sexual orientation, highlighting the relevance of a positive LB identity for CO to parents. Research and clinical implications and directions for future research are discussed.

## Introduction

The experience of coming out (CO) is often a pivotal event in the lives of women who identify as lesbian or bisexual (LB). Prior research on the CO process has mainly focused on lesbian women (Morris, 1997; Jordan and Deluty, 1998; Baiocco et al., 2015; Pistella et al., 2020a), and only a few studies have investigated this phenomenon in bisexual women (Rust, 1993; Morris et al., 2001; Knous, 2006; Balsam and Mohr, 2007; McLean, 2008; Wandrey et al., 2015). Specifically, most studies assessing the CO experience of bisexual people have drawn on monolithic measures of sexual orientation, combining bisexual women and men (Legate et al., 2012; Pistella et al., 2016). Other research has eliminated bisexual people from the analysis altogether or combined bisexual participants with lesbian women and gay men (Helms and Waters, 2016). Only a small number of recent studies have utilized separate measures of sexual orientation, considering LB women separately (Rosario et al., 2001, 2009; Costa et al., 2013; Belmonte and Holmes, 2016). Indeed, recent evidence has demonstrated that the CO process of bisexual people should be examined further, with greater attention paid to gender differences (Costa et al., 2013; Persson and Pfaus, 2015; Wandrey et al., 2015; Pistella et al., 2016; Mathers, 2019; Newcomb et al., 2019).

The CO process is a relevant developmental task for the identity construction of LB women (Mosher, 2001; Rosario et al., 2001; Legate et al., 2012; Baiocco et al., 2015; Scherrer et al., 2015). Specifically, the CO process is defined as the experience of understanding, accepting, and appreciating one’s non-heterosexual identity and starting to reveal one’s sexual orientation to others. Previous research has suggested that CO to family members is a relevant and sometimes crucial developmental task (Savin-Williams, 2003; Willoughby et al., 2008; Scherrer et al., 2015), and that positive parental reactions can increase the child’s well-being and positive development (Ryan et al., 2010). In contrast, negative parental reactions to CO can imply parental rejection and an avoidance of communication on the subject (Baiocco et al., 2015), making the child more likely to develop depression or substance use (Legate et al., 2012; Baiocco et al., 2015) or to even commit suicide (Ryan et al., 2010). Regarding differences in CO to mothers versus CO to fathers, Pistella et al. (2020a) found that lesbian women, compared to gay men, were more likely to hide their sexual orientation from their father and brothers and to come out to their mother, first, followed by their father later on. However, other studies have found no gender differences related to the choice of which parent to CO to first among gay and lesbian young adults (Baiocco et al., 2015) and among lesbian, gay, and bisexual (LGB) adolescents and young adults (D’Augelli et al., 1998; Grov et al., 2006).

Research has also examined potential differences in parents’ reactions to CO, depending on the biological sex of the child, producing inconsistent results. Some studies have found that parents react more negatively to the CO of children of their same biological gender (i.e., fathers react more negatively to the CO of a son and mothers to the CO of a daughter) (D’Augelli, 2006). In line with this result, an Italian study conducted on participants who identified as gay men or lesbian women found that mothers of lesbian daughters were more likely to report a negative reaction to their daughter’s CO than were fathers, in response to their gay son’s CO (Baiocco et al., 2015). It is possible that mothers, especially in the Italian context, may react particularly negatively to their daughter’s CO, because mothers are usually the primary caregivers—responsible for the growth and education of their children—and the mothers of lesbian daughters may feel that they have not been a good female role model, and experience significant anger and guilt as a result (Baiocco et al., 2015). However, other studies and reviews have not confirmed these findings (D’Augelli et al., 2005; Heatherington and Lavner, 2008).

When CO to parents, bisexual people may experience more difficulties than gay or lesbian people (Pistella et al., 2016). Some qualitative studies conducted in the United States have shed light on the tendency of bisexual people—especially bisexual women—to hide and not disclose their sexual orientation, for fear of being rejected (Knous, 2006; Hayfield et al., 2013; Wandrey et al., 2015). These studies have suggested that bisexual women are less likely to come out than lesbian women (Rosario et al., 2001; Koh and Ross, 2006). In general, bisexual women have more negative feelings about their sexual orientation and are less disclosed than lesbian women, and their family members have more negative feelings and reactions to their CO (Balsam and Mohr, 2007; Scherrer et al., 2015; Belmonte and Holmes, 2016).

Another study, conducted in the Italian context, found that bisexual people were less likely to CO to their family than were lesbian women (Pistella et al., 2016); nevertheless, this study did not distinguish between women and men. Bisexual women face particular stigma and prejudice related to their sexual orientation that can inhibit their disclosure not only to family, but also to the sexual minority community (Costa et al., 2013; Roberts et al., 2015; Smalley et al., 2015; Wandrey et al., 2015). For instance, it is a commonly held belief that bisexuality is only a phase and that bisexual women are confused about their sexual orientation. Another common stereotype views bisexual women as promiscuous and unable to commit to a relationship (Eliason, 2001).

### Variables Associated With CO Process in LB Women

A recent review of the literature (Watson et al., 2017) highlighted that the average age of CO amongst bisexual people is higher than that of lesbian women and gay men (Kooden et al., 1979; Rust, 1993). In line with this, a study conducted in the United Studies (Pew Research Center, 2019) found that bisexual individuals were far less likely to CO to significant others in their life (19%), relative to lesbian women and gay men (75%). Also, recent studies conducted in the Italian context have found that the average age of CO amongst bisexual people, regardless of gender, is 18–24 months later than the average age at which gay men and lesbian women CO (Pistella et al., 2020a). To our knowledge, there are no specific data on the average age of CO of Italian LB women.

Certain individual and demographic variables can affect the CO process in LB women, and the literature reports inconsistent results about the impact of these variables on the CO process. For instance, lesbian women have been found to relinquish their religious identification when they CO, considering the abandonment of their prior religion a central aspect of their CO process (Morris, 1997). Similarly, in a qualitative study conducted by Belmonte and Holmes (2016), LB women reported that they hid their sexual orientation when they were with religious people, including family members. Thus, it is plausible to assume that having a religious family and/or living in a traditional or conservative family may discourage LB women from disclosing their sexual orientation, as found in a previous study that did not distinguish between LB women and gay men (Schope, 2002; Maslowe and Yarhouse, 2015; Pistella et al., 2016; Salvati et al., 2018).

Previous studies have suggested that if young people have a traditional/religious or politically right-wing/conservative family of origin (Schope, 2002; Pistella et al., 2016; Salvati et al., 2018), they are less likely to disclose their sexual orientation in an attempt to avoid negative parental reactions (Heatherington and Lavner, 2008; Baiocco et al., 2018a). However, Pistella et al. (2016) did not detect any relationship between religiosity and CO, and Maslowe and Yarhouse (2015) found religiosity to be related to parental acceptance of CO. Again, Pistella et al. (2016) found that higher levels of education were related to a greater likelihood of CO to family members. To our knowledge, there are no specific data on individual and demographic variables that influence the CO process of lesbian and, in particular, bisexual women.

### Benefits and Costs of CO Process

Coming out has also been found to relate to both positive and negative aspects of well-being and positive identity. In particular, sharing one’s sexual orientation with others may support self-integration and social acceptance (Corrigan and Matthews, 2003). Research has also stressed that the CO process is associated with greater life satisfaction (Griffith and Hebl, 2002; Heatherington and Lavner, 2008), self-esteem (Savin-Williams, 1989), emotional relief (Monroe, 2000), and physical and life well-being and job satisfaction (LaSala, 2000; Griffith and Hebl, 2002), as well as reduced anxiety (Monroe, 2000), and the development of a positive sense of self (Rosario et al., 2001). A study by Ryan et al. (2015) found lesbian women to have more positive reactions and to feel more autonomy and satisfaction after CO, relative to gay men. A further study on LB women showed that their disclosure of sexual orientation increased their self-esteem and positive affectivity, decreasing anxiety and enhancing social support (Jordan and Deluty, 1998).

Conversely, other research has shown that CO can be a negative experience, by exposing young people to violence, verbal abuse, and rejection (D’Augelli and Grossman, 2001; Pistella et al., 2020a,b). These negative outcomes may have a significant impact on psychological well-being (Baiocco et al., 2015), especially amongst bisexual individuals, more so than gay and lesbian people (Ross et al., 2018). Bonet et al. (2007) stressed how the CO of LB women is associated with high levels of discrimination and stress and possible negative consequences (D’Augelli and Hershberger, 1993). In particular, CO may lead LB women to be rejected, exposed to verbal and physical violence, and excluded from groups (Rosario et al., 2002).

Koh and Ross (2006) found higher levels of psychological distress and suicidality among LB women compared to heterosexual women. Moreover, it has been shown that LB women may be rejected and disapproved of by family, in addition to peers, with severe consequences for psychological health, including greater stress (Rosario et al., 1996) and substance use (Russell et al., 2002). However, the CO process can also enhance self-esteem by welcoming subjects into the LGB community and solving the conflict related to their developmental task, thereby decreasing depression and anxiety (Crocker and Major, 1989).

The CO process has also been found to be associated with internalized sexual stigma (ISS; Lingiardi et al., 2012)—that is, the internalization of negative feelings, representations, and attitudes toward a non-heterosexual orientation that sexual minority people inflict upon themselves, either consciously or unconsciously (Herek et al., 2009). By preventing self-acceptance of one’s sexual orientation, a high level of ISS can lead young people to hide and deny their orientation (D’Augelli et al., 2002; Lingiardi et al., 2012; Salvati et al., 2018) and consequently come out less to family members (D’Augelli and Grossman, 2001; Chow and Cheng, 2010; Pistella et al., 2016, 2020a,b), friends, and significant others (Lingiardi et al., 2012; Costa et al., 2013; Salvati et al., 2018).

The theoretical framework of the minority stress model can assist our understanding of the negative impacts of stigma, discrimination, expectations of rejection, violence, and ISS, as chronic and psychological stressors for sexual minorities (Meyer, 1995, 2003; Sandfort et al., 2006). Within this framework, it is conceivable that young people with a high level of ISS are less likely to CO (Durso and Meyer, 2013). It is important to note that recent studies have found that minority stress causes more psychological distress for LB women, relative to other sexual minorities, suggesting that there is a need for more in-depth research into minority stress among these populations (Prell and Traeen, 2018; Scandurra et al., 2020).

Recent studies (Riggle et al., 2014; Baiocco et al., 2018b) have investigated positive variables that might promote CO, finding that CO is related to the development of a positive LGB identity, associated with neither positive nor negative attitudes. A positive LGB identity is the result of a multifaceted evolutive process characterized by a progressive combination and integration of feelings, thoughts, and emotions arising from an awareness that one’s sexual orientation can enhance one’s individual, social, and relational functioning (Mohr and Kendra, 2011; Riggle et al., 2014; Rostosky et al., 2018; Petrocchi et al., 2020). Having a positive LGB identity is not equivalent to lacking negative attitudes and feelings about one’s sexual orientation. Indeed, positive LGB identity and negative LGB identity, in terms of ISS, are not opposite poles of the same continuum (Moradi et al., 2009; Petrocchi et al., 2020). A study by Petrocchi et al. (2020) found that ISS was negatively and modestly related to all dimensions of a positive LGB identity. To our knowledge, there is a paucity of studies on the relationship between positive identity and CO process: Some authors have highlighted how positive identity predicts the CO process (Monroe, 2000; Baiocco and Pistella, 2019) while other researchers have described CO as a process that is conducive to personal growth and positive identity (LaSala, 2000; Savin-Williams, 2003). Anyhow, further studies are needed to better understand the nature of this relationship.

### Present Study

Within the theoretical framework of the minority stress model (Meyer, 1995, 2003), and driven by the need for more research on LB women (Pistella et al., 2016; Prell and Traeen, 2018; Scandurra et al., 2020), the present research had three main objectives: (a) to provide descriptive data on CO to parents in a sample of LB women, including the age of first disclosure to mothers and fathers and the quality of the parental reactions; (b) to examine the role of certain sociodemographic variables (i.e., age, sexual orientation, socioeconomic status, education level, political orientation, religiosity and religious education, presence of a stable relationship, family size) in CO to parents; and (c) to explore the relationship between CO to parents, ISS, positive LB identity, and sexual orientation.

Specifically, we predicted that: (H1) women with a higher level of ISS, regardless of their sexual orientation, would be less likely to reveal their sexual orientation to mothers and fathers; (H2) positive LB identity would mediate the impact of ISS on self-disclosure to parents; and (H3) the mediation model would be moderated by sexual orientation. Specifically, we aimed at examining whether sexual orientation (i.e., self-identification as lesbian vs. bisexual) would moderate the path from ISS to CO to mothers, fathers, or both parents. Moderated mediation was supported when the model paths differed as a function of sexual orientation. No specific hypotheses were made regarding the possible moderation effect of sexual orientation.

## Materials and Methods

### Procedures

The original sample consisted of 449 women who had been recruited through online advertisements and an Internet-based survey. The inclusion criteria were: (a) Italian nationality, (b) female biological sex, (c) lesbian or bisexual sexual orientation, (d) aged 40 years or younger, and (d) at least one parent alive. On the basis of these criteria, 6 participants were excluded due to sexual orientation (5 pansexual, 1 asexual), 12 were excluded because they did not indicate that at least one of their parents was alive, and 4 were excluded because they did not complete the entire set of questionnaires. Moreover, the research did not include other sexual orientations (e.g., pansexual or asexual), because prior studies have reported that the CO experience of these sexual minorities differs significantly from that of LB women.

Participation was voluntary and anonymous, and all respondents completed the same set of questionnaires (requiring approximately 10–15 minutes). Informed consent was obtained from all participants, and no compensation was provided for participating in the research. In total, 98% of women who accessed the online survey completed the entire questionnaire. Prior to initiating data collection, the research protocol was approved by the Ethics Commission of the Department of Developmental and Social Psychology, Sapienza University of Rome, Italy. All procedures performed with human participants were conducted in accordance with the ethical standards of the institutional and/or national research committee and with the 1964 Declaration of Helsinki and its later amendments, or comparable ethical standards.

### Participants

The final sample consisted of 427 Italian women who self-identified as lesbian (*n* = 241; 56%) or bisexual (*n* = 186; 44%), with ages ranging from 18 to 40 years (lesbian women: *M* = 27.61, *SD* = 7.19; bisexual women: *M* = 25.23, *SD* = 5.81). Among the lesbian women, 5 (2%) did not have a mother, whereas 23 (9%) did not have a father (bisexual women: 7 [4%] and 12 [7%], respectively). The general level of education was high, with 56 (23%) lesbian women and 55 (30%) bisexual women having at least a university degree, and 142 (59%) lesbian women and 98 (53%) bisexual women having completed secondary school. With respect to socioeconomic status, 78 (32%) lesbian women reported an above average status, whereas 135 (56%) reported an average status (bisexual women: 68 [37%] and 94 [51%], respectively).

### Measures

#### Sociodemographic Variables

Participants completed an identifying form to provide sociodemographic data pertaining to sex, age, sexual orientation, socioeconomic status (0 = poor, 2 = good), education level (0 = high school, 2 = Ph.D., specialization), political orientation (0 = left-wing, 2 = right-wing), and parental situation (parents alive vs. both parents deceased or out of contact). Participants also indicated whether they adhered to any religion, with the dichotomous item “Are you a believer?” (no = 0, yes = 1). Following this, they were asked: “Have you received a religious education?” Here, participants indicated their response using a 5-point Likert scale ranging from 0 (*not at all*) to 4 (*very much*). In addition, participants were asked to indicate whether they were an only child (=0) or whether they had at least one sibling (=1). The presence of a stable relationship was investigated by the following item: “Do you have, at this time, a stable romantic relationship?” The answer modality was dichotomous (no = 0, yes = 1). Finally, participants were asked to report their sexual orientation by indicating one of three possible responses (0 = lesbian woman; 1 = bisexual woman; 2 = other). In cases where “other” was selected, participants had the opportunity to specify their sexual orientation.

#### Disclosure of Sexual Orientation

Each participant was given a list of two significant figures (i.e., mother and father) and asked to indicate whether each figure was aware of their sexual orientation. Three possible responses were provided: “He/she is aware of my sexual orientation,” “He/she is not aware of my sexual orientation,” and “not applicable” (e.g., if a parent was not alive). Participants were also asked to indicate the age of their first disclosure to each parent. Parents’ reactions to the CO were categorized into one of four response options (accepting, tolerant, intolerant, or rejecting). Previous research has also used these measures of CO (D’Augelli et al., 1998; Pistella et al., 2020a,b).

#### Measure of Internalized Sexual Stigma for LB Women–Short Version (MISS-LB; Lingiardi et al., 2012)

The short version of the MISS-LB was used to measure participants’ ISS (Pistella et al., 2016; Baiocco et al., 2018b). The scale was adapted for the current research in order to evaluate such aspects in bisexual women, as well. Example items included: “I would prefer to be heterosexual” and “I do not believe in love between lesbian women or bisexual women.” Participants answered on a 5-point Likert scale ranging from 1 (*I disagree*) to 5 (*I agre*e). A mean score of six items was calculated, with higher scores indicating greater levels of ISS. Cronbach’s alpha was 0.76.

#### Positive LB Identity

The Italian version of the Multifactor LB Positive Identity Measure (LB-PIM; Riggle et al., 2014; Baiocco et al., 2018b) is a 25-item adapted measure designed to assess positive identity in LB women through five dimensions: self-awareness (e.g., “I am more aware of how I feel about things because of my LB identity”), authenticity (e.g., “I am comfortable with my LB identity”), community (e.g., “I feel supported by the LB community”), intimacy (e.g., “My LB identity allows me to be closer to my intimate partner”), and social justice (e.g., “My experience with my LB identity leads me to fight for the rights of others”). Respondents rate each item on a 7-point scale ranging from 1 (*strongly disagree*) to 7 (*strongly agree*). In accordance with previous research (Baiocco et al., 2018b; Pistella et al., 2020a), we used the average total score for the analysis, with a higher score indicating greater positive LB identity. Previous studies using the total score have indicated excellent internal consistency (Pistella et al., 2020a). In the current research, Cronbach’s α for the total score was 0.95.

### Data Analysis

Bivariate and multivariate analyses were conducted using the Statistical Package for the Social Sciences (SPSS 25.0). Sexual orientation differences in relation to CO to parents, ISS, positive LB identity, and other covariates were examined using chi-square tests and univariate analyses of variance (ANOVAs). Kruskal–Wallis *H*-tests were used to reveal differences in parents’ reactions to CO between lesbian women and bisexual women. Point-biserial (when one variable was continuous and one was dichotomous), Pearson (when both variables were continuous), and phi (when both variables were dichotomous) coefficient correlations were calculated to examine the relationships between variables.

Finally, we tested different mediation models using the Process SPSS macro (Hayes, 2009, 2013), and evaluated the direct and mediating effects for statistical significance with bias-corrected bootstrapping (5,000 samples) and 95% confidence intervals (CI). We also examined moderated mediation models to measure the effect of sexual orientation (lesbian vs. bisexual). All continuous variables were standardized to *z*-scores prior to the analysis.

## Results

### Sexual Orientation Differences and Associations Between Key Variables

More women reported that they had CO to their mother (*n* = 289, 69%) than their father (*n* = 203, 52%), regardless of sexual orientation. Descriptive statistics of the measure, differentiated by sexual orientation, are presented in Table 1. Sexual orientation differences were found in relation to CO to mothers, CO to fathers, ISS, and positive LB identity. In particular, more lesbian women had revealed their sexual orientation to both parents, relative to bisexual women. Again, the analyses showed that lesbian women reported lower levels of ISS and higher levels of positive LB identity compared to bisexual women. There were no significant differences between LB women regarding other sociodemographic characteristics, such as SES, educational level, political orientation, religiosity, stable relationship, and family size.

**TABLE 1:** Descriptive of the sample’s characteristics.

	Lesbians (*n* = 241)	Bisexuals (*n* = 186)	Total sample (*n* = 427)	*t/F/χ*^2^	*p*
(1) CO to mother (*yes*)	184 (78%)	105 (59%)	289 (69%)	17.95	< 0.001
(2) CO to father (*yes*)	130 (60%)	73 (42%)	203 (52%)	12.11	< 0.01
(3) Positive LB identity	5.67 (0.98)	5.20 (1.14)	5.47 (1.08)	20.94	< 0.001
(4) Internalized Sexual Stigma (ISS)	1.52 (0.67)	1.71 (0.76)	1.60 (0.71)	3.59	0.01
(5) Age	27.61 (7.19)	25.23 (5.81)	26.57 (6.68)	3.79	< 0.001
(6) SES (*average*)	135 (56%)	94 (51%)	229 (54%)	1.27	0.53
(7) Education level (*high school*)	142 (59%)	98 (53%)	240 (56%)	2.35	0.31
(8) Political orientation (*left wing*)	160 (66%)	138 (74%)	298 (70%)	4.18	0.12
(9) Religiosity (*yes*)	55 (23%)	47 (25%)	102 (24%)	0.35	0.56
(10) Religious education	1.74 (0.91)	1.75 (0.92)	1.75 (0.91)	0.11	0.91
(11) Relationship (*yes*)	139 (58%)	95 (51%)	234 (55%)	1.85	0.17
(12) Family size (*sibling(s)*)	203 (84%)	144 (77%)	347 (81%)	3.20	0.07

*The t/F/χ^2^ it refers to the sexual orientation difference in total sample (LB women). Standard deviations and percentages are in parentheses. Totals for CO to mother and CO to father vary because some women did not have a father or a mother.*

No differences were found between LB women in terms of the figure to whom participants first CO. Paired-sample *t*-tests showed statistically significant differences between the average age of CO to mothers versus the average age of CO fathers, *t*(194) = −4.13, *p* < 0.001, indicating that participants in both groups tended to CO first to mothers. On average, CO to mothers occurred at age 20 (*SD* = 5.54, range = 10–40 years), while CO to fathers occurred at age 22 (*SD* = 5.63, range = 13–40 years).

Participants’ reports of mothers’ and fathers’ CO reactions are reported in Table 2. Overall, paired-sample *t*-tests revealed no significant differences between mothers’ and fathers’ reactions, *t*(53) = −3.53, *p* = 0.001. Similarly, no differences in mothers’ and fathers’ reactions between LB women were found (Table 2). Almost half of the mothers and fathers of both LB women were perceived as tolerant of their daughter’s sexual orientation, but not fully accepting. Amongst lesbian women, 11% of mothers and 11% of fathers were perceived as fully accepting, while bisexual women reported higher frequencies of 15% and 18%, respectively.

**TABLE 2:** Mother and father responses reported by those who had disclosed.

	Mothers’ reactions		Fathers’ reactions	
	Lesbians (*n* = 184)	Bisexuals (*n* = 105)	Lesbians (*n* = 130)	Bisexuals (*n* = 73)
Rejecting	27 (15%)	15 (14%)	15 (12%)	11 (15%)
Intolerant	65 (35%)	29 (28%)	34 (26%)	17 (23%)
Tolerant	71 (39%)	49 (47%)	62 (47%)	32 (44%)
Accepting	21 (11%)	12 (11%)	19 (15%)	13 (18%)
*M*	2.47	2.55	2.65	2.64
*SD*	0.87	0.86	0.88	0.82
Kruskal–Wallis test	*H*(1) = 0.86; *p* = 0.35		*H*(1) = 0.99; *p* = 0.98	

*All the values refer to participants who revealed their sexual orientation. The Kruskal–Wallis test refers to the difference between LB women.*

Table 3 shows correlations among key variables for the overall group of women. There was a significant moderate correlation between CO to mothers and CO to fathers. CO to both parents was negatively correlated with ISS and positively related with positive LB identity and age. Interestingly, CO to mothers was weakly associated with participants who were only children. Finally, CO to both parents, positive LB identity, and ISS were significantly correlated with the presence of a stable relationship.

**TABLE 3:** Correlations between CO to parents, Internalized Sexual Stigma (ISS) and positive LB identity and other variables considered in the present study (*n* = 427).

	1	2	3	4	5	6	7	8	9	10	11	12
(1) CO to mother	1.00											
(2) CO to father	0.66**	1.00										
(3) Positive LB identity	0.27**	0.30**	1.00									
(4) ISS	−0.18**	−0.22**	−0.40**	1.00								
(5) Age	0.17**	0.21**	0.14**	−0.12*	1.00							
(6) SES	0.10*	0.09	−0.12**	0.02	–0.08	1.00						
(7) Education level	0.05	0.05	–0.07	–0.02	0.26*	0.19**	1.00					
(8) Political orientation	–0.05	–0.03	–0.06	0.09	–0.02	–0.01	−0.19**	1.00				
(9) Religiosity	–0.07	–0.07	–0.06	0.10*	0.04	0.01	–0.02	0.18**	1.00			
(10) Religious education	–0.06	–0.06	–0.09	0.17**	0.06	0.05	–0.05	0.08	0.53**	1.00		
(11) Relationship	0.12*	0.10*	0.11*	−0.18**	0.10*	–0.05	0.09	–0.03	–0.02	–0.02	1.00	
(12) Family size	−0.10*	0.02	–0.07	–0.03	0.09	–0.06	–0.03	0.02	0.03	0.02	1.00	1.00

**p < 0.01, *p < 0.05. CO to mother and father (0 = He/she is not aware of my sexual orientation to 1 = He/she is aware of my sexual orientation); SES: socioeconomic status (0 = poor to 2 = good); education level; (0 = high school to 2 = Ph.D., specialization); political orientation (0 = left wing to 2 = right wing); religiosity, religious education, and presence of a stable relationship (0 = no, 1 = yes); family size (0 = only child, 1 = sibling/s).

### CO to Parents, ISS, and Positive LB Identity

To investigate whether the relationship between ISS and CO to parents was mediated by positive LB identity, we tested various mediation models. First, we performed mediation analysis, considering CO to mothers and CO fathers as dependent variables in the same model. Specifically, we tested a mediation model in which ISS was the independent variable, CO to mothers and CO to fathers were the dependent variables, and positive LB identity was the mediator. However, there were no significant findings. Thus, we repeated the analysis with a dummy dependent variable coded as: 0 = those who had CO to only one or to neither parent; and 1 = participants who had CO to both parents. A preliminary chi-square test, *χ*^2^(1) = 12.84, *p* < 0.001, indicated that lesbian women (*n* = 147, 61%) had CO more frequently to both parents compared to bisexual women (*n* = 81, 43%). Previous research has recoded these variables similarly (Pistella et al., 2020a).

**FIGURE 1:**
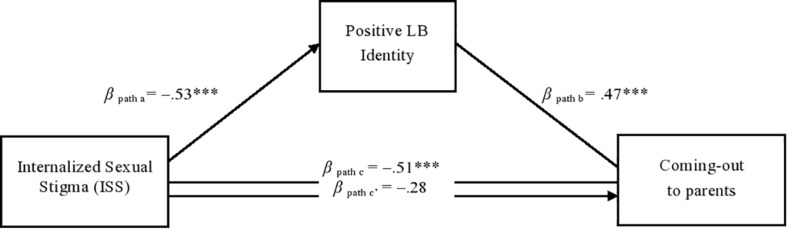
The mediated effect of positive LB identity on the relationship between ISS and CO to parents. ***p < 0.001. All values are beta coefficients. In the ISS and positive LB identity scales, a higher score indicates greater internalized sexual stigma and positive LB identity, respectively. A higher score to the coming out variable indicate more likely to reveal their sexual orientation to both parents. Age, Sexual orientation, SES, education, presence of a stable relationship, and family size were included as covariates.

We then tested a mediation model in which the relationship between ISS and CO to both parents was mediated by positive LB identity. We adjusted our analyses for a number of covariates: age, sexual orientation, socioeconomic status, education level, political orientation, religiosity and religious education, presence of a stable relationship, and family size (only child vs. sibling(s)). The results are presented in Figure 1. When examining the relationship between ISS and CO to both parents, we found a significant direct effect (see β path c in Figure 1; H1). When we entered the mediator in the model, there was a total reduction in the relationship between ISS and CO to parents (see β path c’), providing support for our second hypothesis (H2). The individual paths showed that ISS was negatively related to positive LB identity (β path a), which in turn was positively related to CO to both parents (β path b). ISS and positive LB identity accounted for a significant amount of variance in CO to parents, *R*^2^_Nagelkerke_ = 0.20, *p* < 0.001.

An examination of indirect effects showed that positive LB identity significantly mediated the association between ISS and CO to both parents (bootstrapping estimate = −0.26, SE = 0.08, 95% CI = −0.43, 0.11). Among the covariates considered in the model, only age, β = 0.05, SE = 0.02, *p* = 0.003, and SES, β = 0.45, SE = 0.17, *p* = 0.007, were associated with CO to both parents, while sexual orientation, β = −0.40, SE = 0.22, *p* = 0.06, education level, β = 0.08, SE = 0.15, *p* = 0.58, political orientation, β = 0.04, SE = 0.16, *p* = 0.79, religiosity, β = −0.18, SE = 0.29, *p* = 0.52, religious education, β = −0.10, SE = 0.22, *p* = 0.62, presence of a stable relationship, β = 0.34, SE = 0.21, *p* = 0.10, and family size, β = −0.08, SE = 0.27, *p* = 0.76, were not. We also examined moderated mediation models to verify the effect of sexual orientation as a moderator (lesbian vs. bisexual women), but there were no significant findings. Thus, the last hypothesis (H3) was not supported by the moderated mediation.

In addition, given that a causal relationship was not assumed between ISS and positive LB identity, alternative models were tested using the same key variables with counter pathways (results available upon request). When we considered positive LB identity as the independent variable and ISS as the mediator, the mediation model was not significant (bootstrapping estimate = −0.08, SE = 0.05, 95% CI = −0.18, 0.02). Therefore, we concluded that our original model was the most adequate in describing the association between positive and negative aspects of LB identity and CO to parents.

## Discussion

As there is a paucity of studies in the literature on LB women, the present study sought to investigate the process of CO to parents, focusing on these specific sexual minority populations. Previous research has typically combined bisexual men and women in a single sample (Legate et al., 2012; Pistella et al., 2016); however, “diversity within the LGB community should not be overlooked,” as stated by Costa et al. (2013), (p. 241). Furthermore, prior research has not typically differentiated between CO to mothers and CO to fathers (Costa et al., 2013; Pistella et al., 2016), nor has it generally considered CO to both parents, as performed here. The present study found no differences between LB women regarding either the age of CO to parents or fathers’ and mothers’ reactions to the disclosure. Nonetheless, more lesbian women had CO to both mothers and fathers relative to bisexual women, and both LB women were more likely to CO first to mothers and, within the following 2 years, to fathers, in line with a recent Italian study conducted by Pistella et al. (2020a). These results are consistent with previous studies, suggesting that bisexual women are more likely than lesbian women to hide and not disclose their sexual identity for fear of being rejected (Knous, 2006; Hayfield et al., 2013; Wandrey et al., 2015).

The innovative aspects of the present study relate to the investigation of the role of ISS and positive LB identity in inhibiting or promoting LB women’s CO to both parents. First, the results showed that lesbian women had lower levels of ISS and higher levels of positive LB identity than bisexual women, suggesting that bisexual women may comprise a more vulnerable group, exposed to higher levels of sexual stigma. The literature stresses that bisexual people must cope with more negative prejudice and stigma than lesbian women and gay men (Eliason, 1997), and that bisexual people, in general, have to manage stigma and rejection even from the sexual and gender minority community (Mohr and Rochlen, 1999; Friedman et al., 2014). It is also important to consider that this research was conducted in the Italian context, which is characterized by a sexist and heteronormative culture and a high level of sexual prejudice (Lingiardi et al., 2012; Baiocco and Pistella, 2019); this, in itself, may lead LB women—especially bisexual women—to develop a high level of ISS and a low level of positive identity (Baiocco et al., 2018b; Petrocchi et al., 2020).

An even more interesting result is the detection of the hypothesized mediation effect of a positive LB identity in the relationship between ISS and CO to both parents, within both LB women. Specifically, ISS was found to be negatively related to a positive LB identity, which, in turn, was associated with CO to both parents. The mediation model was tested whilst controlling for the effects of demographic variables, but only age and socioeconomic status were found to have a significant positive effect on CO to both parents. Due to the impossibility of assuming a causal relationship between ISS and positive LB identity, we tested an alternative model in which ISS was the mediator variable between positive LB identity and CO to both parents. However, this model did not yield a significant mediation effect of ISS, highlighting that our hypothesized model should be preferred. The present findings suggest the significant role of a positive LB identity in reducing the negative effect of ISS on the CO process to parents (Riggle et al., 2014; Rostosky et al., 2018; Petrocchi et al., 2020).

Although bisexual women reported higher levels of ISS and lower levels of LB positive identity than did lesbian women, data from the mediation model suggests that the development of a positive LB identity could be a protective factor for both LB women. As suggested by Petrocchi et al. (2020), a positive LB identity should be fostered in LB people, because it can protect them from negative and discriminative experiences—particularly in the Italian heterosexist context. Furthermore, a positive LB identity can promote resilience and adaptive functioning in LB women, leading to greater psychological well-being.

The present findings have implications that extend beyond social science research, because they may also be useful for clinical practice with LB women, who have unique therapeutic needs (Scherrer, 2013; Baiocco and Pistella, 2019). It is important that future studies evaluate not only dimensions related to psychological distress (e.g., ISS), but also positive dimensions (e.g., the development of a positive LB identity), especially among the younger generations of sexual minority people. Moreover, future studies on the CO process should consider the mediating role of a positive LB identity in the relationship between ISS and CO.

Regarding clinical implications, the findings could advise mental health professionals on which variables they should take into account when working with LB women to promote a positive CO process and adaptation to family contexts (Baiocco and Pistella, 2019). Clinicians should be aware that bisexual women may face additional prejudice and suffer from higher levels of ISS, relative to lesbian women; however, it is important that clinicians focus on strengthening and promoting a positive LB identification in both LB women. In most clinical settings, psychotherapists working with LB clients aim at reducing ISS and helping clients to alleviate the negative effect that minority stress can have on CO process, working on negative emotions associated with stigmatizing experiences and developing a positive self-image (Scandurra et al., 2020).

Again, our results suggest that clinicians and mental health professionals should focus their attention—in different phases of the work and according to patients’ needs—on strengthening and supporting a positive LB identity. This, in turn, might encourage LB women to disclose their sexual identity to their parents. Thus, it is important that clinicians and mental health professionals working with LB women aim at increasing their clients’ resilience, positive identity, coping strategies, self-awareness, authenticity, intimacy, and resources and strengths, in line with the guidelines of affirmative therapy with sexual minority people (O’Shaughnessy and Speir, 2018). LB women who are supported in developing a positive identity may be more likely to CO, because they are more resilient to any prejudice, stigma, and rejection that may arise in response to their disclosure. Therefore, CO can provide an opportunity for personal growth and the development of inter- and intrapersonal resources, which can generate more social support for LB women, as they are no longer hiding their sexual identity (Riggle and Rostosky, 2012; Kwon, 2013).

In terms of practical implications in the social context, the present results suggest that educational, work, and cultural contexts should promote positive and visible models of LB women. Indeed, early intervention in these environments may prevent young LB women from becoming discouraged, which may lead them to conceal their sexual orientation in family, cultural, and social contexts due to a fear of being discriminated against. Programs and events should be developed to combat sexism and homophobia, such as campaigns to support sexual and gender minority rights (Baiocco and Pistella, 2019). Over recent years, Italian society has faced several challenges. We are aware that much work remains to be done to improve sexual minority acceptance in the Italian context; however, we are motivated to continue our efforts to promote the positive identity and well-being of sexual and gender minority people.

Despite its innovative aspects, the study also has some limitations. First, we used a convenience sample that was not representative of the general population, and we did not focus explicitly on any differences that may exist on the basis of age, race, class, or gender. Second, we used self-report questionnaires, which are subject to a common method bias (Podsakoff et al., 2003). Moreover, the study was conducted in Italy—a conservative, family-oriented, and heterosexist country, in which young people are typically more involved in family dynamics than are youth in other Western societies (Baiocco et al., 2015). Thus, future cross-cultural studies should be conducted to confirm our mediation model in other cultural contexts. Finally, it was not possible to infer causal relationships among the variables, due to the correlational nature of data. Future longitudinal studies may be conducted to more deeply test the possible effects of ISS and positive LB identity on the process of CO to parents.

The present study contributes deeper knowledge of the differences between LB women during the development of their sexual orientation, highlighting the relevance of a positive LB identity in influencing CO to parents. Moreover, the finding that ISS impacts CO to both parents via a positive LB identity in both LB women supports further investigation into these populations within the field of CO. Nevertheless, considering the scarcity of data on the CO process of LB women, the present study constitutes an important step forward in our understanding of sexual minority women’s experiences with their parents.

## Data Availability Statement

The dataset generated for this study is available upon request to the corresponding author.

## Ethics Statement

The studies involving human participants were reviewed and approved by ethics committee of the Department of Developmental and Social Psychology, Sapienza University of Rome. The patients/participants provided their written informed consent to participate in this study.

## Author Contributions

RB and JP contributed to conceptualization of the project and creation of the research design and instruments, executed the study, assisted with the data analyses, and wrote the manuscript. MM and JP collaborated with designing and writing the study. RB collaborated in writing and editing the final manuscript. All authors read and approved the final manuscript.

## Conflict of Interest

The authors declare that the research was conducted in the absence of any commercial or financial relationships that could be construed as a potential conflict of interest.
